# A case report of liver failure caused by *Tetrastigma hemsleyanum* Diels et Gilg: A rare case report and literature review

**DOI:** 10.1097/MD.0000000000042059

**Published:** 2025-03-28

**Authors:** Yan Liu, Xia Zhou, Qiong-Fen Wang, Ming-Wei Liu

**Affiliations:** a Department of Gastroenterology, The People’s Hospital of Lincang City, Lincang, Yunnan, China; b Department of Human Resources, Dali Bai Autonomous Prefecture People’s Hospital, Dali, China; c Department of Gastroenterology, Dali Bai Autonomous Prefecture People’s Hospital, Dali, Yunnan, China; d Department of Emergency, Dali Bai Autonomous Prefecture People’s Hospital, Dali, China.

**Keywords:** case report, diagnosis, liver failure, *Tetrastigma hemsleyanum* Diels et Gilg, treatment

## Abstract

**Rationale:**

*Tetrastigma hemsleyanum* Diels et Gilg is a traditional Chinese medicinal plant recognized for its therapeutic properties, which include heat-clearing, toxicity-eliminating, anti-inflammatory, pain-relieving, blood circulation-promoting, blood stasis-resolving, wind-dispersing, and phlegm-resolving actions. It is predominantly used in clinical settings to manage inflammatory disorders, such as febrile convulsions, hepatitis, snakebites, and cellulitis. To date, no documented cases of liver failure attributable to *T hemsleyanum* Diels et Gilg have been reported.

**Patient concerns:**

A 50-year-old female patient presented with symptoms of xanthochromia, tea-colored urine, fatigue, and anorexia following a three-day course of *T hemsleyanum* Diels et Gilg. Liver function tests revealed elevated alanine aminotransferase (1386 U/L) and AST (405 U/L) levels.

**Diagnoses:**

Liver failure induced by *T hemsleyanum* Diels et Gilg was diagnosed.

**Interventions:**

The patient received treatment that included discontinuation of hepatotoxic drugs, administration of N-acetylcysteine for liver protection, nutritional support, and correction of acid-base and electrolyte imbalances.

**Outcomes:**

After 11 days of liver-protective and nutritional therapy, significant improvements in the patient’s condition were noted. The symptoms of xanthochromia and tea-colored urine subsided, and liver function markers decreased markedly, returning to near-normal levels. No further complaints of discomfort were reported, and the patient was discharged with prescribed medications for follow-up.

**Lesson:**

The possibility of liver failure following the use of *T hemsleyanum* Diels et Gilg should be considered. When patients present with symptoms such as fat intolerance, xanthochromia, poor appetite, nausea, and dark urine after consuming this herb, liver function tests should be promptly conducted to exclude the possibility of drug-induced liver injury. *T hemsleyanum* Diels et Gilg has the potential to induce liver failure. Liver-protective measures, including nutritional support, proved to be effective in managing the condition.

## 1. Introduction

Drug-induced liver injury (DILI) refers to hepatic damage resulting from a variety of prescription and over-the-counter chemical drugs, traditional Chinese medicines (TCMs), natural substances, biological agents, health products, dietary supplements, their metabolites, and even excipients.^[1]^ DILI is responsible for more than half of all cases of acute liver failure in the United States^[2]^ and is the leading cause of acute liver failure in both the United States and Europe. A study conducted by Reuben et al in the United States demonstrated that, among patients with acute liver failure caused by DILI, the survival rate was only 23% in the absence of liver transplantation; similar outcomes were observed in Sweden.^[3]^ Research has indicated^[4]^ that the annual incidence of DILI in the general population of China is no <24 per 100,000 residents, a figure that exceeds rates in Western countries, thereby highlighting DILI as a significant clinical issue in China. According to Chinese literature, the 5 primary categories of drugs leading to DILI are Chinese medicines, antibiotics, antipyretic analgesics, antituberculosis medications, and cardiovascular drugs.^[4,5]^ In China, Chinese medicines or herbs and dietary supplements (26.81%) and antituberculosis drugs (21.99%) account for the majority of DILI cases.^[4]^

With the growing utilization of TCM and continuous advancements in monitoring techniques, adverse reactions, particularly herb-induced liver injury (HILI), have become increasingly prevalent.^[6]^ Common herbs implicated in DILI include *Sedum aizoon L.*, *Periploca forrestii*, *Polygonum multiflorum*, *Tripterygium wilfordii*, *Dioscorea bulbifera*, *Xanthium sibiricum*, and *Cinnabar*.^[6]^ *Tetrastigma hemsleyanum* Diels et Gilg, a member of the *Vitaceae* family, is utilized in both its fresh form and as dried tubers. It is characterized by a mildly bitter taste, a neutral or cool nature, and is considered nontoxic. The herb is known for its properties in clearing heat and toxicity, reducing inflammation and pain, and alleviating wind and phlegm.^[7]^ Referred to as the “plant antibiotic,” *T hemsleyanum* Diels et Gilg is predominantly found in the southern regions of China, including Zhejiang, Jiangxi, Fujian, Guangxi, and Guizhou provinces.^[7]^ It is extensively used in clinical practice to treat conditions such as pediatric high fever convulsions, rheumatism, dysentery, bronchitis, pneumonia, pharyngitis, hepatitis, and viral meningitis,^[7]^ with minimal documented toxic side effects. However, there have been no reported cases of liver failure resulting from the use of *T hemsleyanum* Diels et Gilg.

## 2. Case report

### 2.1. Ethics statement

Written informed consent was obtained from each individual(s) for publication of potentially identifiable images or data included in this article. Written informed consent was obtained from the participants (s) for publication of this case report. This study was reviewed and approved by the local ethics committee of the Lincang People’s Hospital. The procedures were performed in accordance with the Helsinki Declaration of 1975, revised in 2000.

### 2.2. Medical history

A 50-year-old female patient presented with xanthochromia and tea-colored urine, along with fatigue and anorexia, following a three-day course of *T hemsleyanum* Diels et Gilg. Liver function tests conducted at a local hospital revealed signs of liver failure. Subsequently, the patient was admitted to the Department of Gastroenterology at Lincang People’s Hospital, Yunnan Province, on February 30, 2024, for further evaluation and management.

### 2.3. Past medical history

The patient has no history of diabetes mellitus, cardiovascular or cerebrovascular diseases, nor any significant disorders involving the pulmonary, hematological, or endocrine systems. There is no reported history of infectious diseases, alcohol consumption, or hepatitis. Additionally, no prior trauma, surgical interventions, blood transfusions, or allergies have been documented. The patient’s vaccination history remains unclear.

### 2.4. Physical examination

The patient’s temperature was 36.3 °C, pulse rate was 87 beats per minute, respiratory rate was 20 breaths per minute, and blood pressure was 126/80 mm Hg. The patient appeared to be in poor general condition, exhibiting severe xanthochromia of the skin and sclera. Clear breath sounds were auscultated bilaterally, with no evidence of dry or moist rales. Cardiac percussion did not reveal any enlargement of the heart border. The heart rate was 87 beats per minute, with a regular rhythm, and no pathological murmurs were detected across all valve auscultation areas. The abdomen was flat and soft, with tenderness noted around the periumbilical region but without rebound tenderness or muscle rigidity. Both the liver and spleen were not palpably enlarged. Murphy’s sign was negative, and shifting dullness was also absent. Bowel sounds were present at a rate of 4 times per minute. Moreover, no edema was observed in the lower extremities.

### 2.5. Laboratory data

On February 20, 2024, the complete blood count indicated a white blood cell count of 5.27 × 10^9^/L, neutrophils at 68.4%, lymphocytes at 8.3%, a red blood cell count of 4.71 × 10^12^/L, hemoglobin levels at 152 g/L, and a platelet count of 242 × 10^9^/L. Liver function tests demonstrated alanine aminotransferase (ALT) at 1386 U/L, aspartate aminotransferase (AST) at 907 U/L, γ-glutamyl transpeptidase at 202 U/L, total bile acid at 255.3 μmol/L, albumin at 33 g/L, total bilirubin (TBIL) at 243.6 μmol/L, direct bilirubin at 138.7 μmol/L, and indirect bilirubin (IBIL) at 104.9 μmol/L. Coagulation parameters revealed a prothrombin time of 15.2 s, an international normalized ratio of 1.62, activated partial thromboplastin time of 49.7 seconds, thrombin time of 20.4 s, fibrinogen at 5.04 g/L, and a D-dimer level of 0.63 mg/L. Blood ammonia was measured at 61 μmol/L, and viral hepatitis markers were negative. The Roussel Uclaf Causality Assessment Method (RUCAM) score was calculated as 7.

### 2.6. Pathological examination data

**Figure 1.**
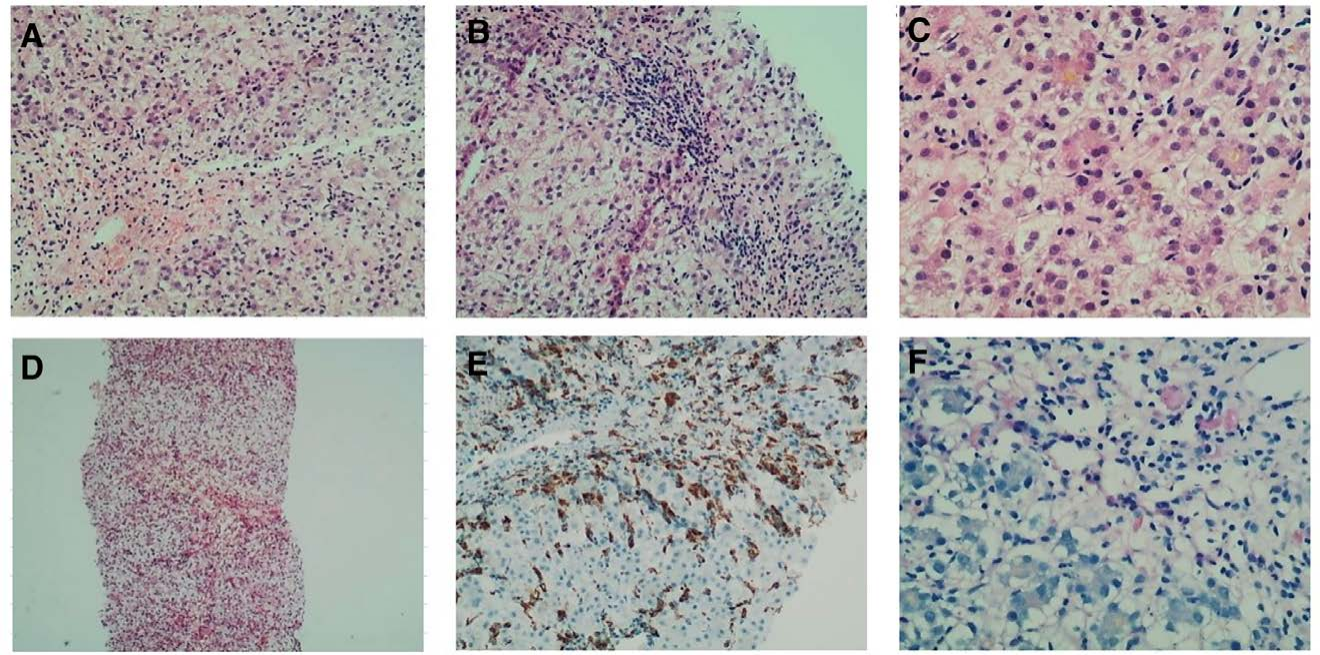
Pathological changes in liver biopsy upon admission. (A) Hepatocyte confluent necrosis, hemorrhagic edema, and inflammatory cell infiltration; (B) bridging necrosis; (C) small bile thrombus with surrounding hepatocytes arranged in a rosette pattern; (D) sirius red staining; (E) CD68; (F) diastase-resistant (DPAS) staining.

The liver biopsy performed on February 27, 2024, demonstrated a disrupted hepatic lobular architecture, with infiltration of the portal area by lymphocytes, occasional plasma cells, and histiocytes. Partial injury to the bile ducts, along with degeneration and necrosis of the bile duct epithelium, was observed. No significant abnormalities were noted in the interlobular veins and arteries. Hepatocytes exhibited swelling, cholestasis, and feathery degeneration, with occasional signs of steatosis. Canalicular dilatation accompanied by bile plugs was also observed, and rosette formations were evident. Extensive focal necrosis was present throughout the lobules, with confluent necrosis primarily localized in zone 3 and areas of focal bridging necrosis. The proliferation of sinusoidal Kupffer cells was noted, and necrosis of the central vein was identified in some regions. Sirius Red staining did not reveal any increase in fibrous tissue. CD68 staining demonstrated extensive proliferation and activation of sinusoidal Kupffer cells, with aggregation of interstitial histiocytes. Diastase-resistant staining was positive for Kupffer cells (Fig. 1).

### 2.7. Imaging data

**Figure 2.**
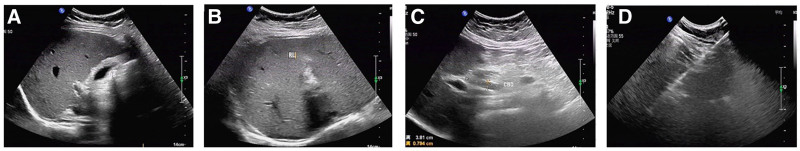
Abdominal ultrasound findings upon admission.

**Figure 3.**
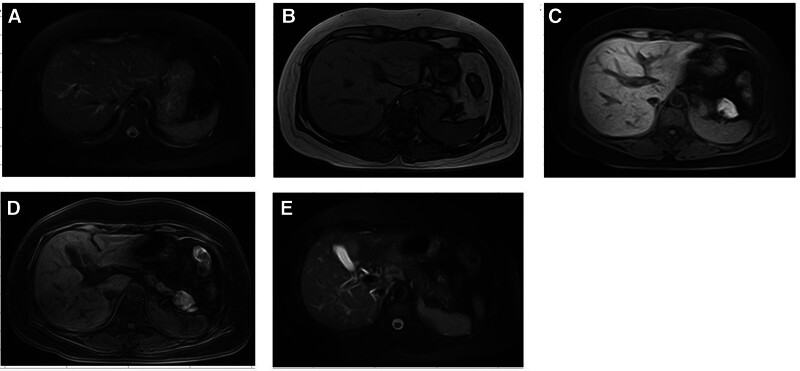
Abdominal MRI findings upon admission. MRI = magneticresonance imaging.

Abdominal ultrasound revealed no significant abnormalities in the liver, gallbladder, spleen, or pancreas (Fig. 2). Magneticresonance imaging of the abdomen demonstrated normal liver size, with the appropriate proportions of all lobes. During the arterial phase, patchy enhancement was noted in the right lobe of the liver, while uniform enhancement in the venous and delayed phases was consistent with normal liver parenchyma, suggesting a localized perfusion anomaly. No focal abnormal signals were identified in other regions of the liver. The spleen appeared of normal size and showed no signs of enlargement. Furthermore, no dilation was observed in the intrahepatic or extrahepatic bile ducts, common hepatic duct, common bile duct, or the main pancreatic duct. The gallbladder was found to be of normal size (Fig. 3).

### 2.8. Diagnosis and treatment

The diagnosis of liver failure induced by *T hemsleyanum* Diels et Gilg was established based on the patient’s medical history, symptoms, signs, liver function tests, and pathological examination results. After the exclusion of autoimmune hepatitis, systemic immune disorders, and liver injuries caused by hepatitis viruses, cytomegalovirus, and Epstein-Barrvirus, the diagnosis was confirmed. The primary course of treatment involved the immediate cessation of the hepatotoxic agent and the administration of hepatoprotective medications, including 2 g/d of reduced glutathione and 80 mg/d of N-acetylcysteine (NAC), along with supportive nutritional therapy. Throughout the treatment process, dynamic monitoring of liver function indicators and the patient’s clinical status was conducted. On the 4th day of treatment, repeat liver function tests revealed notable decreases in ALT, AST, TBIL, and IBIL levels (Table 1). Following an additional 14-day treatment period, the patient’s symptoms showed substantial improvement, with the resolution of both skin and scleral jaundice. Subsequent liver function tests indicated further reductions in ALT and AST levels, with TBIL and IBIL returning to within normal ranges (Table 1). After evaluation by the attending physician, the patient was discharged with prescribed medications for continued treatment.

**Table 1:** Changes in liver function of patients before and after treatment.

	At admission	4d after treatment	7d after treatment	14d after treatment	14d after discharge
ALT (U/L)	1386	104.91	212	76	57
AST (U/L)	907	149	67	45	43
TBIL (µmoI/L)	243.61	241.3	154.6	57.1	52.9
DBIL (µmoI/L)	138.7	141.5	81.7	28	18.6
IBIL (µmoI/L)	104.91	99.8	72.9	29.1	34.3

ALT = alanine aminotransferase, AST = aspartate aminotransferase, DBIL = direct bilirubin, IBIL = indirect bilirubin, TBIL = total bilirubin.

### 2.9. Follow-up after treatment

At the two-week follow-up visit following discharge, the patient reported no discomfort. Repeated laboratory tests indicated that ALT and AST levels had normalized, and both TBIL and IBIL were within the reference ranges (Table 1). The patient was advised to refrain from using *T hemsleyanum* Diels et Gilg and any TCM formulations containing this herb.

## 3. Discussion

Most instances of HILI are considered idiosyncratic, with only a few reports of intrinsic liver damage.^[8]^ Although *P multiflorum* and *T wilfordii* are acknowledged for their intrinsic hepatotoxic potential, the majority of herbal remedies induce HILI through idiosyncratic hepatotoxic mechanisms. Hepatotoxic effects have been documented for various herbs, such as *Psoralea corylifolia*, *Dictamnus dasycarpus*, *S aizoon L.*, *T wilfordii*, *Scutellaria baicalensis*, *Bupleurum chinense*, *Melia azedarach L.*, *Dichroa febrifuga*, *Albizia julibrissin*, and *D bulbifera*.^[9]^ However, to date, no cases of liver failure caused by *T hemsleyanum* Diels et Gilg have been reported.

The factors contributing to liver injury induced by TCM can be categorized as follows: (1) TCM-related factors: first, the chemical composition and pharmacological properties of TCM are highly complex, with some herbs exhibiting both therapeutic and toxic effects, capable of either treating diseases or causing liver damage.^[10]^ Examples include *Artemisia argyi*, *B chinense*, and *Rheum palmatum*. Second, certain herbs previously considered nontoxic have been identified as having hepatotoxic properties, such as *Trichosanthes kirilowii*, *P multiflorum*, *D bulbifera*, and *Cassia angustifolia*.^[10]^ Third, the confusion caused by different herbs sharing the same name or by similar herbs having different names can result in accidental poisoning, as seen with *Stephania tetrandra* and*Akebia quinata*. Fourth, the variability in the quality of TCM, stemming from differences in sourcing, cultivation, processing, and storage, leads to inconsistencies in both efficacy and toxicity. Acute liver injury is often linked to overdose, allergic reactions, or injectable forms, while chronic liver injury typically results from toxin accumulation due to prolonged oral use.^[10]^ (2) Patient-related factors: First, liver injury may arise from improper use, self-medication, or excessive reliance on certain toxic herbs, folk remedies, secret formulas, and patent medicines, with the latter causing DILI from prolonged use or overuse.^[11]^ Second, patient-specific factors, such as age, health status, and individual susceptibility, influence the risk of liver injury. Vulnerable populations, including children, the elderly, pregnant women, and those with compromised health, are more prone to DILI.^[11,12]^ The mechanisms underlying liver injury induced by *T hemsleyanum* Diels et Gilg remain unstudied.

The active constituents in TCM serve a pivotal function in determining both their therapeutic and toxicological effects. Improper or excessive use of herbal medicines can lead to detrimental outcomes. TCMs often contain a variety of potentially harmful compounds, which can be categorized according to their chemical structures into alkaloids, glycosides, diterpenes, lactones, anthraquinones, phytotoxic proteins, and heavy metals.^[13,14]^ These toxic agents have the potential to exacerbate hepatotoxicity. For instance, anthraquinone derivatives, such as 1,8-dihydroxy-3-hydroxymethyl anthraquinone, isolated from medicinal plants like rhubarb and cassia seed, have been shown to induce liver damage in animal models.^[14]^ The incidence of liver injury linked to alkaloids and glycosides is notably higher compared to other chemical groups.^[15,16]^ Current investigations suggest that *T hemsleyanum* Diels et Gilg contains several bioactive compounds, including flavonoids, phenolic acids, terpenes, fatty acids, and polysaccharides, which may contribute to its potential hepatotoxicity. However, to date, no cases of liver injury associated with *T hemsleyanum* Diels et Gilg have been documented.

Currently, the diagnosis of DILI remains challenging due to the absence of distinctive clinical and pathological features. Consequently, a comprehensive assessment of the patient’s history, biochemical parameters, pathological findings, and imaging results is essential for an accurate diagnosis. The RUCAM scale has proven to be a valuable and reliable tool for determining the likelihood of DILI, owing to its quantitative, structured, and transparent nature.^[17]^ The primary biochemical markers used in clinical practice for diagnosing DILI include ALT, ALP, AST, and TBIL.^[18]^ Histopathological analysis is indispensable in differentiating DILI from other hepatic conditions, particularly in cases where the cause of liver dysfunction is unclear. In the present case, the patient developed jaundice of both the skin and sclera after the administration of *T hemsleyanum* Diels et Gilg, with substantial elevations in liver enzyme levels, including ALT, AST, and TBIL, indicative of hepatic injury.The RUCAM score of 9 further confirmed a high likelihood of drug-induced liver damage, and pathological findings from the liver biopsy revealed severe acute drug-induced hepatitis. Consequently, the diagnosis of liver injury resulting from *T hemsleyanum* Diels et Gilg was established.

In the majority of DILI cases, patients show significant improvement or even complete recovery following the discontinuation of the offending drugs, although a small subset may progress to acute liver failure.^[19]^ Pharmacological interventions for DILI typically focus on mechanisms such as free radical scavenging, antioxidation, hepatocyte protection, stabilization of cell membranes, detoxification, transaminase reduction, and immune modulation.^[20]^ It has been demonstrated that NAC serves as a targeted treatment for acetaminophen-induced liver injury. The therapeutic effect of NAC is primarily attributed to its ability to scavenge a variety of free radicals in the body, with earlier administration yielding more favorable outcomes.^[20]^ Magnesium isoglycyrrhizinate exerts its effects through multiple mechanisms, including the scavenging of free radicals, inhibition of inflammatory mediators like leukotrienes and prostaglandins, and modulation of T cell activation. These actions contribute to the reduction of transaminase levels, resolution of jaundice, anti-inflammatory effects, membrane stabilization, hepatoprotection, and immune regulation.^[20]^ Both high-dose and low-dose magnesium isoglycyrrhizinate have been shown to provide significant therapeutic efficacy and safety in DILI management.^[20]^ As a result, NAC combined with magnesium isoglycyrrhizinate was chosen as the treatment regimen for this patient.

## 4. Strengths and limitations

### 4.1. Strengths

*T hemsleyanum* Diels et Gilg has been implicated in cases of liver failure, and the cessation of this herb, coupled with hepatoprotective therapy using NAC and magnesium isoglycyrrhizinate, has demonstrated therapeutic efficacy.

### 4.2. Limitations

The exact pathogenesis of IDILI remains poorly understood, due to its relatively low incidence. Despite *T hemsleyanum* Diels et Gilg being associated with liver failure, the number of documented cases is still limited, indicating the necessity for large-scale, multicenter studies. The precise mechanism through which *T hemsleyanum* Diels et Gilg induces liver failure remains uncertain and warrants further investigation. Given the herb’s complex array of active constituents, additional studies are required to elucidate which specific compound or combination of compounds contributes to liver injury.

## 5. Conclusion

*T hemsleyanum* Diels et Gilg has the potential to induce liver failure. When symptoms such as fat intolerance, jaundice, reduced appetite, and nausea manifest following the consumption of this herb, DILI attributed to *T hemsleyanum* Diels et Gilg should be considered. In such cases, immediate liver function tests should be conducted, and hepatoprotective treatment should be promptly administered.

## Acknowledgments

We would like to thank the Home for Researchers editorial team (www.home for researchers.com) for the English language editing.

## Author contributions

**Conceptualization:** Yan Liu, Xia Zhou, Qiong-Fen Wang.

**Data curation:** Xia Zhou, Qiong-Fen Wang, Ming-Wei Liu.

**Formal analysis:** Xia Zhou.

**Funding acquisition:** Yan Liu, Ming-Wei Liu.

**Investigation:** Yan Liu, Qiong-Fen Wang.

**Methodology:** Xia Zhou, Qiong-Fen Wang.

**Project administration:** Yan Liu, Ming-Wei Liu.

**Resources:** Yan Liu, Xia Zhou, Qiong-Fen Wang.

**Supervision:** Yan Liu, Ming-Wei Liu.

**Software:** Qiong-Fen Wang

**Validation:** Xia Zhou, Qiong-Fen Wang.

**Visualization:** Yan Liu, Xia Zhou, Qiong-Fen Wang.

**Writing – original draft:** Ming-Wei Liu.

**Writing – review & editing:** Ming-Wei Liu.
